# Atezolizumab plus modified docetaxel-cisplatin-5-fluorouracil (mDCF) regimen versus mDCF in patients with metastatic or unresectable locally advanced recurrent anal squamous cell carcinoma: a randomized, non-comparative phase II SCARCE GERCOR trial

**DOI:** 10.1186/s12885-020-06841-1

**Published:** 2020-04-25

**Authors:** Stefano Kim, Bruno Buecher, Thierry André, Marine Jary, François-Clément Bidard, François Ghiringhelli, Éric François, Julien Taieb, Denis Smith, Christelle de la Fouchardière, Jérôme Desramé, Emmanuelle Samalin, Aurélie Parzy, Nabil Baba-Hamed, Olivier Bouché, David Tougeron, Laëtitia Dahan, Farid El Hajbi, Marion Jacquin, Magali Rebucci-Peixoto, Laurie Spehner, Véronique Vendrely, Dewi Vernerey, Christophe Borg

**Affiliations:** 1 Department of Oncology, University Hospital of Besançon, 3 Boulevard Alexander Flemingn, F-25030 Besançon, France; 2 Hôpital Nord Franche Comté, Montbéliard, France; 3 Clinical Investigational Center, CIC-1431, University Hospital of Besançon, Besançon, France; 4 INSERM, Unit 1098, University of Bourgogne Franche-Comté, Besançon, France; 5 Groupe Coopérateur Multidisciplinaire en Oncologie (GERCOR), Paris, France; 6 Fédération Francophone de Cancérologie Digestive (FFCD), Paris, France; 7 Institut Curie, Paris, France; 8 Sorbonne Université and Hôpital Saint Antoine, Paris, France; 9 Centre Georges-François Leclerc, Dijon, France; 10 Centre Antoine-Lacassagne, Nice, France; 11 Hôpital Européen Georges-Pompidou, Paris, France; 12 Centre Hospitalier Universitaire de Bordeaux, Bordeaux, France; 13 Centre Léon Bérard, Lyon, France; 14 Hôpital Privé Jean Mermoz, Lyon, France; 15 Institut du Cancer de Montpellier, Montpellier, France; 16 Centre François Baclesse, Caen, France; 17 Groupe Hospitalier Paris Saint-Joseph, Paris, France; 18 Centre Hospitalier Universitaire de Reims, Reims, France; 19 Centre Hospitalier Universitaire de Poitiers, Poitiers, France; 20 Centre Hospitalier Universitaire La Timone, Marseille, France; 21 Centre Oscar Lambret, Lille, France; 22 Cancéropôle Grand-Est, Strasbourg, France; 23 Methodology and Quality of Life in Oncology Unit, University Hospital of Besançon, Besançon, France

**Keywords:** Anal carcinoma, Advanced, Atezolizumab, Chemotherapy, Immunotherapy, Docetaxel

## Abstract

**Background:**

Modified docetaxel, cisplatin, and 5-fluorouracil (mDCF) regimen has become a new standard for the treatment of metastatic or unresectable locally advanced recurrent squamous cell carcinoma of the anus (SCCA) after demonstrating improved efficacy (12-month PFS of 47%) in the Epitopes-HPV02 trial. Antibodies targeting the checkpoint inhibitor (CKI) programmed cell death protein-1 (PD1) have demonstrated the efficacy as monotherapies in second-line treatment of SCCA. The aim of this study is to evaluate the combination of atezolizumab and mDCF as first-line chemotherapy in a non-comparative multicentre randomized phase II study of advanced SCCA patients.

**Methods:**

Patients with chemo-naive advanced histologically proven SCCA, metastatic or unresectable locally advanced recurrence, and Eastern Cooperative Oncology Group-performance status (ECOG-PS) < 2 will be eligible. The primary endpoint is a 12-month PFS rate. Using one-arm non-parametric survival with unilateral alpha type I error of 5% and a statistical power of 80%, the upper critical value for the 12-month PFS rate is 47% to reject H0. Assuming 5% lost to follow-up, 99 patients will be randomized on a 2:1 basis, 66 to the experimental arm (arm A, mDCF plus atezolizumab) and 33 to the standard arm (arm B, mDCF). In both arms, 8 cycles of mDCF will be administered. In arm A, patients receive mDCF with a fixed dose of atezolizumab (800 mg every 2 weeks) and are followed up to 1 year. Secondary endpoints are overall survival, PFS, response rate, safety, health-related quality of life, and an extensive biomarker programme and its correlation with the treatment efficacy.

**Discussion:**

Although the Epitopes-HPV02 trial has changed long-lasting prognosis of patients with SCCA in advanced stage disease, more than 50% of patients will progress at 12 months. The purpose of the SCARCE trial to establish the addition of atezolizumab to mDCF as a new standard in this rare disease. Associated biomarker studies and the control arm could contribute to better understanding of the potential synergic and tumour resistance mechanisms in SCCA.

**Trial registration:**

NCT03519295.

## Background

Squamous cell carcinoma of the anus (SCCA) is still considered as a rare disease accounting for less than 3% of all gastrointestinal tumours [1], but the annual incidence rate is increasing worldwide mostly due to its association with human papillomavirus (HPV) infection, predominantly with the genotype HPV-16 [2]. Approximately 15% of patients are diagnosed at an advanced stage [3] and 25–40% will experience disease progression after curative intent chemoradiotherapy (CRT) for localized disease [4]. In patients with non-resectable local recurrences or with distant metastases, the systemic chemotherapy is the standard approach.

Recently, the modified docetaxel, cisplatin, and 5-fluorouracil (mDCF) combination chemotherapy regimen has demonstrated high efficacy, with good tolerance in a phase II Epitopes-HPV02 trial of 69 SCCA patients [5] and thus has become the first validated chemotherapy regimen in advanced SCCA. The objective response rate (ORR) was as high as 90.9%, with complete response of 45.5%. The median PFS was 11 months and the median overall survival (OS) was still not reached at a median follow-up of 33.4 months [6]. In addition, mDCF demonstrated significantly lower toxicity compared to the standard DCF (Grade III/IV toxicity rate of 53% vs 83%) while maintaining the same efficacy [5]. More recently, these encouraging results were externally validated with the updated data from the Epitopes-HPV02 trial including 51 SCCA patients. The ORR reached 85.1% (40 of 47 evaluable patients), with a complete response of 34% (16 of 47 patients). The median PFS was 12.7 months (95% CI, 11.2–34.5) and the median OS was 50.2 months (95% CI, 21.4–120.0) [6]. In addition, the translational research program demonstrated the ability of DCF to induce clearance of HPV oncoprotein-related DNA in patient blood (HPV circulating tumour DNA [ctDNA]), which was correlated with long-term remissions. In SCCA, 95% of cases are related to HPV, mostly to genotype HPV16 (89%) [7]. HPV-negative SCCA tumors are rare, frequently TP53-mutated, and have a worse prognosis [8, 9]. In HPV-related cancers, HPV viral genomes are usually integrated into the tumour cell genome or episomal DNA. Droplet-digital PCR (ddPCR) detects and quantifies HPV ctDNA at baseline in almost all advanced stage patients, and in 0% of healthy controls as well as women treated for HPV16-associated high grade intraepithelial neoplasia [10]. In the Epitopes-HPV02 trial, HPV ctDNA was detected at baseline in 91% of patients. After treatment with DCF, residual HPV ctDNA was present in 39% of patients and was highly predictive of disease progression (HR = 5.5; *p* < 0.001) and OS (HR = 7.0; *p* = 0.02) [11], validating the interest of HPV ctDNA in routine clinical practice in advanced SCCA patients [12].

Another biomarker of interest in this disease setting is telomerase reverse transcriptase (TERT). TERT is an active enzyme during embryonic foetal development, but not in non-self-renewing somatic cells. Our previously data supports the use of TERT as an HPV-related antigen in SCCA [5]. We observed that TERT-specific immune responses significantly influence OS in SCCA patients after DCF chemotherapy (*p* = 0.040) in comparison with HPV-specific immune response (*p* = 0.09) [5].

Besides, the carboplatin and paclitaxel (CP) combination regimen has demonstrated an improved safety compared to cisplatin and 5-fluorouracil (5-FU; CF) in the InterAACT randomized phase II trial of 90 SCCA patients. Although grade III/IV adverse events rate was similar (71–74%) between both arms, there were significantly less severe adverse events with CP (36%) compared to CF (62%). However, this trial has failed to meet its primary endpoint; ORR was 59 and 57.1% with CP and CF, respectively [13].

Despite these practice-changing results, 53% of patient treated with DCF and approximately 85% of patients treated with CP will present disease-progression at 12 months; and at date, there is no validated chemotherapy regimen in second-line. The programmed cell death protein-1 immune checkpoint (PD1) and its ligand (PD-L1) are particularly relevant target candidate for immunotherapy in SCCA patients, based on the prominent role of PD-1/PD-L1 in HPV-driven immune-evasion [14–16]. Two prospective non-controlled trials have shown the interest of immunotherapies in this disease [17, 18]. Nivolumab and pembrolizumab, two antibodies targeting PD1, had demonstrated antitumour activity in refractory patients to at least one line of chemotherapy. Objective responses were observed in 20% of patients, with estimated 12-month PFS and OS rates of 20 and 48%, respectively [17, 18].

The association of an anti-PD1/PD-L1 with chemotherapy seems promising. The combination of different chemotherapy regimens and an anti-PD1/PD-L1 were feasible with improved survival in first-line advanced small-cell and non-small-cell lung cancers [19–21]. In anal carcinoma, the Epitopes-HPV02 trial showed that the mDCF regimen was feasible with 53% of grade 3–4 adverse-events, with no grade 4 non-haematological toxicities, and without febrile neutropenia. Given that mDCF may enhance antitumour immune response, it was recommended as an interesting candidate to be evaluated as a backbone chemotherapy for immunotherapy combinations in SCCA [5].

Therefore, we decided to associate a PD1/PD-L1 inhibitor to the mDCF-based chemotherapy regimen to improve the efficacy with higher rate of long-lasting progression-free survivors. The associated extensive ancillary biomarker studies of tumour tissues and peripheral blood samples will provide a unique opportunity to assess a potential synergy mechanism between mDCF and checkpoint inhibitor (CKI) and will help to improve our knowledge about underlying resistance mechanisms.

## Methods and analysis

SCARCE is a randomized, non-comparative, multicentre phase II trial to evaluate the combination of atezolizumab and the mDCF regimen in advanced SCCA patients. The study was developed by the “National Institute of Health and Medical Research (INSERM), Unit 1098”, and “Clinical Investigational Center (CIC) 1431”. It is coordinated and sponsored by the GERCOR Medical Oncology group and supported by the PRODIGE collaborative oncologic group. The data management is undertaken by the “Methodology and Quality of Life Unit in Oncology”* of the University Hospital of Besançon. The trial is registered on the clinicaltrials.gov (NCT03519295) and is conducted in accordance with the Declaration of Helsinki and the Good Clinical Practice (GCP).

* http://www.umqvc.org/en/index.html

### Study objectives

*The primary endpoint* is to evaluate the observed PFS rate at 12 months from the initiation of DCF in patients with metastatic or unresectable locally advanced recurrent SCCA. PFS is defined as the time from randomization to progression (evaluated by the RECIST criteria version 1.1) or death from any cause, whichever occurred first.

*The secondary endpoints* are:

- To evaluate OS,
- To evaluate PFS,
- To evaluate health-related quality of life (HRQoL),
- To evaluate ORR,
- To evaluate the tolerance of DCF in in association with atezolizumab,
- To evaluate the predictive value of HPV-specific and telomerase-specific T cell responses monitored before and after treatment,
- To analyse HPV, p53, and neo-antigens genotypes and their correlation with the treatment efficacy,
- To investigate the impact of peripheral immune system status (Treg, CD4+ polarization, myeloid-derived suppressor cells [MDSC], T-cell exhaustion) on clinical outcomes and HPV/telomerase specific immunity,
- To investigate the prognostic value of tumour-infiltrating lymphocytes and PD-L1 expression,
- To explore the correlation of both peripheral CD4+ anti-telomerase immunity and PDL1 immunohistochemistry with PFS,
- To characterize the predictive value of soluble biomarkers (e.g. soluble PD-L1) and plasmatic HPV DNA monitoring,
- To evaluate the correlation between neo-antigen burden and survival at 12 months.

### Patient selection

The study population consists of patients with histologically proven SCCA at advanced stage defined as:

- Stage IV disease with distant metastases, or
- Locally advanced recurrence after CRT, non-eligible for salvage surgery due to the extension of the disease.

Patients should have an Eastern Cooperative Oncology Group (ECOG) Performance **...**

(ECOG-PS) of 0 or 1 and adequate organ functions. The inclusion and exclusion criteria are listed in Table 1.

**Table 1:** Main inclusion and exclusion criteria of the trial

***Inclusion criteria*** ➢ Histologically proved, metastatic or unresectable locally advanced recurrent SCCA, ➢ Age ≥ 18 years, ➢ ECOG-PS of 0 or 1, ➢ Signed written informed consent. ***Exclusion Criteria*** *Non-eligibility to clinical trials:* ➢ Previous received chemotherapy for metastatic disease, ➢ Previous received cisplatin, except for concomitant CRT, ➢ Previous chemotherapy taxanes or another spindle poison, ➢ Previous received anti-tumour immunotherapy (HPV vaccination is allowed), ➢ Previous radiotherapy within 28 days of randomization (14 days if radiotherapy of bone metastases), ➢ Diagnosis of additional malignancy within 3 years prior to randomization with the exception for curatively treated basal cell carcinoma of the skin and/or curatively resected in situ cervical or breast cancer, ➢ Any medical or psychiatric condition of disease, which would make the patients inappropriate for entry into this study, ➢ Current participation in a study of an investigational agent or in the period of exclusion, ➢ Pregnancy, breast-feeding, or absence/refusal of adequate contraception for fertile patients, *Non-eligibility to chemotherapy:* ➢ Inadequate organ functions: uncontrolled cardiac condition, known cardiac failure, unstable coronaropathy, respiratory failure, and Chronic Obstructive Pulmonary Disease (COPD), ➢ Diabetes with vascular or neurovascular complications, ➢ Pre-existent peripheral neuropathy, ➢ HIV-positive with CD4+ count under 400 cells/mm^3^, ➢ Active hepatitis B or C virus (HBV or HCV) infection, ➢ Active tuberculosis, ➢ Concomitant treatment with CYP3A4 inhibitor like ritonavir, indinavir, or ketoconazole, etc. (Replacement by another drug before randomization, whenever is possible, is allowed), ➢ Known hypersensitivity or contraindication to any of the study chemotherapy drugs (taxanes, cisplatin, 5-fluorouracil), ➢ Uncontrolled infection or another life-risk condition, ➢ Known hearing impairment that contraindicates cisplatin administration, ➢ Inadequate laboratory values: creatinine clearance (CrCl by Modification of Diet in Renal Disease [MDRD] formula) < 60 ml/min, neutrophil count < 1500 /mm^3^, platelets < 100,000/mm^3^, bilirubin 2.5 x upper limit of normal (ULN), aspartate transaminase (AST)/alanine transaminase (ALT) 2.5 x ULN or 5 x ULN with liver metastasis, ➢ Patient with known dihydropyridine dehydrogenase (DPD) deficiency or history. *Non-eligible to immunotherapy:* ➢ Any immunosuppressive therapy (i.e. corticosteroids > 10 mg of hydrocortisone or equivalent dose) within 14 days before the planned start of study therapy, ➢ Active autoimmune disease that has required a systemic treatment in past 2 years (i.e. corticosteroids or immunosuppressive drugs). Replacement therapy (e.g. thyroxine, insulin) is allowed, ➢ Active or history of autoimmune disease or immune deficiency, including, but not limited to, myasthenia gravis, myositis, autoimmune hepatitis, systemic lupus erythematosus, rheumatoid arthritis, inflammatory bowel disease, antiphospholipid antibody syndrome, Wegener granulomatosis, Sjögren syndrome, Guillain-Barré syndrome, or multiple sclerosis, ➢ Patients with a history of autoimmune-related hypothyroidism who are on thyroid replacement hormone are eligible for the study, ➢ Patients with controlled Type 1 diabetes mellitus who are on an insulin regimen are eligible for the study, ➢ Patients with eczema, psoriasis, lichen simplex chronicus, or vitiligo with dermatologic manifestations only (e.g., patients with psoriatic arthritis are excluded) are eligible for the study provided all of following conditions are met: o Rash must cover < 10% of body surface area, o Disease is well controlled at baseline and requires only low-potency topical corticosteroids, o No occurrence of acute exacerbations of the underlying condition requiring psoralen plus ultraviolet A radiation, methotrexate, retinoids, biologic agents, oral calcineurin inhibitors, or high potency or oral corticosteroids within the previous 12 months, ➢ Prior allogeneic bone marrow transplantation or prior solid organ transplantation, ➢ Known active central nervous system metastases and/or carcinomatous meningitis. Subject with previously treated brain metastases and with radiological and clinical stability are allowed, ➢ Previously received an anti-PD1, anti-PDL1, or anti-CTLA4 agent, ➢ Known hypersensitivity or allergy to Chinese hamster ovary cell products or any component of atezolizumab formulation, ➢ History of colorectal inflammatory disease, ➢ History of idiopathic or secondary pulmonary fibrosis (history of radiation pneumonitis in the radiation field fibrosis is permitted), or evidence of active pneumonitis requiring a systemic treatment with 28 days before the planned start of study therapy, ➢ Major surgical procedure other than for diagnosis within 4 weeks prior to initiation of study treatment, or anticipation of need for a major surgical procedure during the course of the study, ➢ Severe infection within 4 weeks prior to initiation of study treatment.	

### Treatments

#### Atezolizumab

Patients in the experimental arm (arm A) receive atezolizumab every 2 weeks for a total of 24 cycles at a fixed dose of 800 mg as 60 min IV infusion before mDCF, for 8 first cycles followed by atezolizumab monotherapy. If treatment is well tolerated at first cycle, atezolizumab perfusion can be reduced to 30 min in the following cycles. No dose adjustment is required. No premedication treatment is required before infusion of atezolizumab.

#### mDCF

Patients in both arms receive 8 cycles of mDCF (docetaxel 40 mg/m^2^ day 1, cisplatin 40 mg/m^2^ day 1 and 5-FU at 1200 mg/m^2^/over 2 days) every 2 weeks.

Given the low-risk (< 10%) of febrile neutropenia with the mDCF protocol, support of granulocyte colony-stimulating factor (G-CSF) for 4 days after mDCF administration is recommended (day 4- day 7) only as secondary prophylaxis.

### Study description (Fig. 1)

#### Therapeutic seqssssuence

- Initial assessments will be performed at baseline, within 28 days prior to the first administration of treatment.

**Fig. 1:**
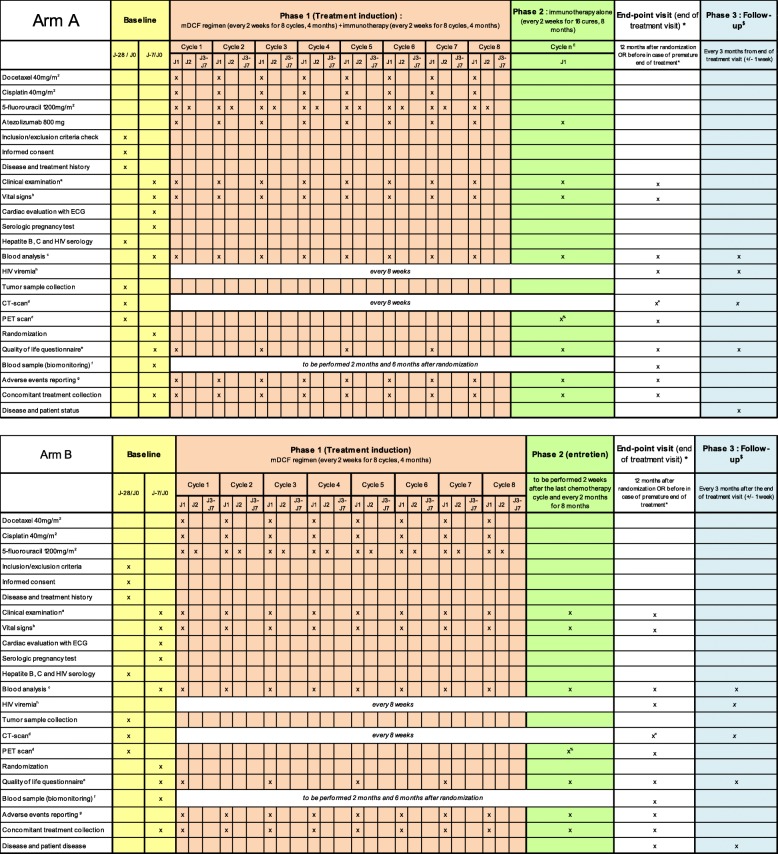
Schedule of enrolment, interventions, and assessments. a Clinical examination: height (at baseline only), weight, and ECOG-PS. b Vital signs: pulse, blood pressure and body temperature. c Blood analysis: complete blood count including red blood cells, haemoglobin, haematocrit, lymphocytes, white blood cells and differentials and platelets, blood electrolytes, bicarbonates, glycemia, proteinemia, albumin, blood urea nitrogen, creatinine, creatinine clearance (MDRD), calcium, magnesium, AST, ALT, gamma-GT, conjugated and total bilirubin, ALP, TSH, LDH, and C-reactive protein. d PET-scan at baseline, at first visit of phase 2 and at the end-point visit (except in case of early progression). CT-scans and PET-scans will be collected for a central review. e The EORTC-QLQC30 questionnaire: every second chemotherapy cycles in phase 1, and every 2 months during phase 2 until the end-point visit. f Blood samples for biomonitoring: 1 EDTA tube of 6 ml (plasma) and 8 EDTA tubes of 6 ml (PBMC). g According to the NCI-CTCAE guidelines version 4.03. h Only in HIV-positive patients. £ Immunotherapy alone: to be continued 1 cycle every to weeks up to a maximum of 12 months from the randomization date. * The end-point visit: to be performed 12 months after randomization or 4 weeks after the last chemotherapy cycle in case of premature end of treatment (for toxicity, progression, or patient or physician decision). $ The follow-up visit: to be performed every 3 months from the end-point visit to a patient death or at least 3 years after randomization

- Patients will be randomized via the electronic case report form (eCRF) and allocated to a treatment on a 2:1 basis into one of the two arms:

- Arm A (66 patients): 8 cycles of the mDCF regimen every 2 weeks in association with atezolizumab administrated every 2 weeks for 12 months.
- Time-frame (TF) 1: 8 cycles of mDCF + atezolizumab (4 months).
- TF 2: 16 cycles of atezolizumab alone (8 months).
- TF 3: follow-up every 3 months for 2 years
- Arm B (33 patients): 8 cycles of the mDCF regimen every 2 weeks.
- TF 1: 8 cycles of mDCF (4 months).
- TF 2: follow-up every 2 months (8 months).
- TF 3: follow-up every 3 months for 2 years. 
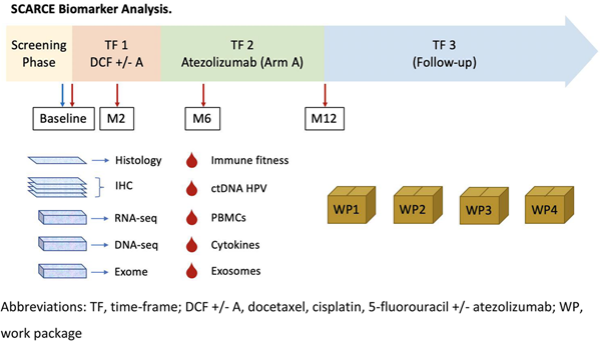

#### Evaluation, laboratory tests, and follow-up

- Computed tomography (CT-scan) will be planned at baseline and every 8 weeks until 12 months (or disease progression) from randomization (TF 1 and 2) and every 12 weeks thereafter (TF 3) in both arms,
- Positron emission tomography (PET) scan will be performed at baseline, at the end of mDCF treatment, and at 12 months after randomization (in absence of disease progression),
- Surgery and/or palliative radiotherapy of residual metastatic sites are allowed according to the Investigator’s centre practices after week 20,
- The end of treatment visit will be performed 12 months after randomization or 4 weeks after the last cycle of treatment if treatment is stopped prematurely (progression, toxicity, or decision of physician or patient),
- Follow-up every 3 months after the end of treatment visit until patient death or at least 3 years after the randomization date.

#### Randomization

Once the patient’s consent is obtained, the Investigator will confirm that all required radiological and biological procedures were performed within schedule before randomization.

Patients will be randomized in a 2:1 ratio to receive mDCF and atezolizumab (Arm A) or mDCF (Arm B).

The randomization will be open following the minimization technic (hazard compound: 0.8) with stratification factors as follows:

- Age (65 < years vs ≥ 65 years),
- Synchronous (metastases at the time of diagnosis) vs metachronous (metastases after starting a treatment for a localized disease) vs locally advanced unresectable disease and no evidence of metastases.

The attribution of the treatment arm will be performed centrally.

Randomization will be performed based on an eCRF form.

### Quality of life assessment

Health-related quality of life (HRQoL) EORTC QLQ-C30 questionnaire will be collected:

- At inclusion, before randomization,
- Every 2 cycles of chemotherapy in TF 1 (mDCF),
- Every 2 months in TF 2 until the end of treatment visit,
- At the end of treatment visit,
- Every 3 months during follow-up in TF 3, until patient death, or at least 3 years after the randomization date.

The EORTC QLQ-C30 questionnaire is validated in French [22]. It consists of 30 items measuring five functional scales (physical, role, emotional, cognitive, and social functioning), a global health status, financial difficulties, and eight scales of symptoms (fatigue, nausea and vomiting, pain, dyspnoea, insomnia, appetite loss, constipation, and diarrhoea). One score is generated per dimension and standardized on a 0 to 100 scale in order that a high score reflects a high GHS, functional and symptomatic level.

### Biological sample collection

Four blood samples will be collected at:

- Sample N°1: At inclusion visit before the first treatment.
- Sample N°2: At 2 months from randomization.
- Sample N°3: At 6 months after randomization.
- Sample N°4: At the end-point visit.

Biomonitoring Blood Samples: seven 6 ml ethylenediamine tetra-acetic acid (EDTA) tubes and two 4 ml EDTA tubes will be collected for analysis.

### Tumour samples

Tumour samples obtained by biopsies or surgery at diagnosis will be centralized for translational research program.

### Data management

For each patient enrolled in the study, the Investigators must document all required data in the corresponding source documents. These data must then be entered into eCRF, which will be accessible only by authorized persons via secured web connection. One eCRF will be created for each patient. The investigator has the responsibility for its completion, proof reading, as well as its approval after the final verification for the authenticity and accuracy of all entered data. The Monitor, who is mandated by the Sponsor, will ensure that the study is conducted in accordance with the GCP guidelines and all applicable local laws and that the rights, security, and well-being of the patient are respected. The Monitor will perform source document verification and validation and request clarification to ensure the accuracy, completeness, and reliability of data. The Investigator guarantees the Sponsor or its representative direct access to source documents. Throughout the study, data electronically captured via eCRF will be regularly checked for consistency, and queries on data clarification will be generated through eCRF. At the end of the data handling process, a data review meeting will be held to prepare the database lock. After database lock, data will be transferred into SAS format to produce statistical analyses.

### Statistical considerations

#### Planed number of patients to include

This study is a 2:1 randomized non-comparative multicentre two-arm phase II study.

The Arm B will serve as calibration arm that the populations in the two arms are similar and to validate the H0 hypothesis in Arm A: no statistical comparison is planned between the two arms. Results acquired in Arm B will also allow the development of a translational investigation to identify potential biomarker for atezolizumab and the mDCF combination in HPV-related diseases and to generate reliable hypothesis for biomarker study.

Then for experimental arm (arm A) we consider the following hypotheses:

- H0: a PFS rate at 12 months of 35% is uninteresting,
- H1: a PFS rate at 12 months of 50% is expected.

Using one arm non-parametric survival with unilateral alpha type I error of 5 and 80% statistical power 62 patients will be required to in 2 years with a 12-month follow-up. The Upper Critical Value for the 12-month PFS rate is 47% to reject H0.

Given 5% lost to follow-up, 66 patients should be randomized in the experimental arm (Arm A) and according to a ratio 2:1, 33 patients into the standard arm (Arm B), for a total of 99 patients.

#### Modality of analysis

The primary analysis will be made on modified intention-to-treat (mITT) population, i.e. including all evaluable randomized patients regardless of their eligibility and treatment received. The results will be reported according to the randomized treatment.

A final statistical plan and a specific statistical plan dedicated to HRQoL analyses will be written before data frozen.

The mITT population will be used for the analyses of all efficacy endpoints. mITT2 population will be used for HRQoL analyses.

The qualitative variables for each modality will be described using numbers and percentages (in relation to the effective). The quantitative variables will be described using the medians, averages, standard deviations, extreme values, and interquartile. For each variable, the number of missing data will be specified. The confidence intervals at 95% (95% CI) will be reported.

The 12-month rate and the median PFS with their 90% CI will be estimated using the Kaplan-Meier method (73). The PFS rates at specified time points will be estimated from the Kaplan-Meier curve and the standard errors by the Greenwood formula. The log-log transformation will be used to compute CIs.

OS and its 95% CI will be calculated between the date of randomization and the date of death from any cause. It will be estimated according to by the Kaplan Meier method. Alive patients or lost to follow-up at the time of the analysis will be censored at the date of last follow-up.

In an exploratory analysis, a hazard ratio (HR) will be estimated using a univariate Cox model with its 90% CI. Independent factors associated with OS (types of metastases, response to the treatment, HRQoL, etc.) will be explored using univariate and multivariate Cox models.

All analyses of HRQoL data will be done on mITT2 population (population mITT1 with at least on HRQoL data available).

HRQoL scores will be described according to the treatment arms with the means (standard deviation) and medians (range Min-Max).

### Study schedule (Fig. 1)

#### Baseline

To be performed within 28 days prior to the initiation of treatment:

- Inclusion/exclusion criteria check, informed consent collection, and HIV serology,
- Clinically relevant medical/surgical history and associated therapies data collection,
- CT-scan and positron emission tomography (PET)-scan (if PET-scan performed more than 28 days prior to the initiation of treatment, it could be validated by the Coordinator considering the accessibility to PET-scan in given centre),
- Tumour sample collection: tumour sample realized in routine will be collected,

To be performed within 7 days prior to the initiation of treatment:

- Clinical evaluation: weight, ECOG-PS, vital signs (pulse, blood pressure, and body temperature),
- Blood analysis: complete blood count including red blood cells, haemoglobin, haematocrit, lymphocytes, white blood cells differentials, platelets, blood electrolytes, bicarbonates, glycemia, protein, albumin, blood urea nitrogen, creatinine, creatinine clearance (CrCl) (by Modification of Diet in Renal Disease [MDRD]), calcium, magnesium, aspartate transaminase (AST), alanine transaminase (ALT), gamma-glutamyl transpeptidase (G-GTP), conjugated and total bilirubin, alkaline phosphatase (ALP), thyroid stimulating hormone (TSH), lactate dehydrogenase (LDH), C-reactive protein (CRP), amylase, lipase
- HIV, HCV, and HBV serology,
- HIV viremia for HIV+ patients,
- Serologic pregnancy test for women with childbearing potential,
- Cardiac evaluation including electrocardiogram (ECG),
- Blood sample n°1: one 6 ml EDTA tube and two 4 ml EDTA tubes (for plasma isolation and freezing) and six 6 ml EDTA tubes (for peripheral blood mononuclear cell [PBMC] storage that should be sent to Besançon Biomonitoring Platform at room temperature, cf. laboratory manual),
  If all eligibility criteria are verified, randomization of the patient is done via the eCRF and a treatment arm is allocated to the patient.

Assessment before each chemotherapy cycle:

Including before cycle 1.

Blood analysis (platelets) done every week until 28 days after treatment induction and in case of fever or sign of infection, or evocative sign of an enterocolitis.

A time windows of +/− 3 days is allowed for chemotherapy cycle.

- Clinical evaluation: weight, height, ECOG-PS, vital signs (pulse, blood pressure and body temperature),
- Blood analysis (within 72 h prior the visit): complete blood count including red blood cells, haemoglobin, haematocrit, lymphocytes, white blood cells and differentials and platelets, blood electrolytes, bicarbonates, glycemia, protein, albumin, blood urea nitrogen, creatinine, CrCl (MDRD), calcium, magnesium, AST, ALT, G-GTP, conjugated and total bilirubin, ALP, TSH, LDH, CRP, amylase, lipase,
- Serologic pregnancy test for women with childbearing potential before each treatment administration,
- HRQoL evaluation with EORTC QLQ-C30 questionnaire: every 2 cycles (cf. Flowchart),
- Adverse events report according to NCI-CTCAE guidelines v 4.03,
- Serious adverse event/Adverse events of special interest report,
- Concomitant treatment collection,
  Assessment to be performed every 8 weeks from randomization to the end of treatment visit:
- CT-scan every 2 months in order to determine the impact of DCF with/without atezolizumab treatment in each patient,
- HIV viremia for HIV-positive patients.
  Biomonitoring: Blood sample n°2, 3: to be taken respectively at 2 and 6 months from the date of randomization: one 6 ml EDTA tube and two 4 ml EDTA tubes (for plasma isolation and freezing), and six 6 ml EDTA tubes (for PBMC storage that should be sent to Besançon Biomonitoring Platform at room temperature, cf. laboratory manual).

### End of trial

This study will end once all 99 recruited patients will complete 3 years of follow-up or die, whichever comes first. The patient can withdraw at any moment and this decision dose no need any justification. The patients will pursue the standard medical care.

Premature discontinuation of the study for the following reasons: patient’s decision, severe adverse event, protocol deviation, loss to follow-up, or death must be declared.

### Monitoring and safety

An Independent Data and Safety Monitoring Committee (IDMC) represented by a multidisciplinary team, including physicians from relevant medical disciplines and biostatisticians, is implemented to:

- ✓ - Ensure the safety and well-being of the patients exposed to study treatment through an ongoing review of safety data,
- ✓ - Assure the highest integrity of the trial operation and performance,
- ✓ - Evaluate ongoing safety data to detect the possibility of an unfavourable early treatment risk.
- In particular the combination of atezolizumab and mDCF will be closely followed.
- IDMC’s responsibilities are:
- ✓ -To review safety data on an ongoing basis,
- ✓ - To recommend premature discontinuation if there is strong evidence that the investigational medicinal products are harming patients,
- ✓ - To make recommendations regarding modification of the study if there is strong evidence that such change would substantially contribute to the well-being of patients.

An IDMC meeting will take place within 10 days following an accomplishment of 2 months of treatment in the first 10 patients. Safety result and opinion of the committee will be sent to the ANSM (French Health Products Safety Agency) within 10 days after tolerance analyses. Additional meetings will be performed in any event of a signal that may affect the patient safety.

The IDMC will independently make its recommendations for continuation or termination of the trial to the appropriate Sponsor contact. The IDMC will maintain records of all its meetings and activities related to the study. These records will remain confidential until completion of the final analysis and only then will be forwarded to the Sponsor for appropriate archival.

The IDMC members will be aware of the data management and quality control procedures in order to assure timely compliance, accuracy, and completeness.

## Discussion

Historically, prognosis of SCCA patients with advanced disease was poor with no prospectively validated chemotherapy regimen. The combinations of CF or CP were recommended based on retrospective analyses demonstrating the median PFS of 5.8–7.0 months and a 5-years overall survival (OS) rate less than 20% [6–8].

Docetaxel, a microtubule-stabilizing agent, exerts cytotoxic functions by blocking dividing cells in G2/M phase, leading to apoptosis. Docetaxel-based chemotherapy can modulate anti-tumour immune responses inducing calreticulin, a damage-associated molecular patterns associated to the immunogenic cell death [23].

Another possible effect of this treatment might be the depletion of immunosuppressive cells, sustaining the potential restoration of effective tumour immunity [24]. We have previously shown promising results with the addition of docetaxel to DCF in our Epitopes-HPV01 cohort study [9]. Four patients (out of eight) presented a long-lasting remission, providing a clinical rational for a prospective multicentre phase II Epitopes-HPV02 trial of 66 SCCA evaluable patients [25]. The trial confirmed the efficacy of this combination and settled the mDCF regimen as a new standard first-line chemotherapy [5]. Moreover, severe toxicities were halved with the mDCF regimen compared to the standard DCF. The immunomonitoring analysis of the Epitopes-HPV01 and Epitopes-HPV02 studies demonstrated that, i) MDSC play a major prognostic role in advanced SCCA patients in first-line, ii) DCF is capable to deplete MDSC and to induce an anti-tumour immune activity [5, 26]. Hence, considering those abilities and the favourable tolerance profile of mDCF, this regimen has been established as a good chemotherapy-based backbone candidate for a combination with immune checkpoint inhibitors [5].

After a long period without progress in advanced SCCA patients, there are now encouraging results with DCF in first-line and with CKIs in second or later lines with long-lasting responders, supporting the synergic effect between these treatments. Therefore, the evaluation of the DCF and an anti-PD1/L1 combination is warranted and will hopefully continue to improve prognosis of patients with this disease. The SCARCE study is the first trial to evaluate the combination of a chemotherapy regimen and CKI in advanced SCCA patients. These results might provide clinical evidence that CKI enhances long-term remission rate achieved by DCF-based chemotherapy and, if positive, could establish a new standard of care in this setting.

## Data Availability

Not applicable.

## Abbreviations

CRT: Chemoradiotherapy
CDDP: Cisplatin
CF: Cisplatin and 5-fluorouracil
CKI: Checkpoint inhibitor
CP: Carboplatin and paclitaxel
CR: Complete response
CTLA-4: Cytotoxic T-lymphocyte-associated protein 4
DCF: Docetaxel, cisplatin, and 5-fluorouracil
ECOG-PS: Eastern Cooperative Oncology Group-performance status
eCRF: Electronic case report form
G-CSF: Granulocyte colony-stimulating factor
HPV: Human papilloma virus
HR: Hazard ratio
IDMC: INDEPENDENT Data and Safety Monitoring Committee
mDCF: Modified DCF, docetaxel 40 mg/m^2^ day, CDDP 40 mg/m^2^ day and 5-FU at 1200 mg/m^2^/day for 2 days, every 2 weeks
MDSC: Myeloid-derived suppressor cells
ORR: Objective response rate
OS: Overall survival
PD1/PD-L1: Programmed cell death protein-1 immune checkpoint/ PD ligand
PFS: Progression-free survival
QoL: Health-related quality of life
SCCA: Squamous cell carcinoma of the anus
sDCF: Standard DCF - docetaxel 75 mg/m^2^/day, CDDP 75 mg/m^2^ and 5FU at 750 mg/m^2^/day for 5 days, every 3 weeks
TF: Timeframe
Treg: Regulatory T cells
